# Increasing TET Expression and 5‐Hydroxymethylcytosine Formation by a Carbocyclic 5‐Aza‐2′‐deoxy‐cytidine Antimetabolite

**DOI:** 10.1002/anie.6265286

**Published:** 2026-06-21

**Authors:** Maike Däther, Elsa Peev, Annika Fröhlich, Binje Vick, Sogol Fatourechi, Gilles Gasparoni, Matthias Heiss, Corinna C. Pleintinger, Emmanuel Asu Bisong, Hans Hurmiz, Davide Guglielminotti, Yasmin V. Gärtner, Tina Aumer, Karsten Spiekermann, Jörn Walter, Irmela Jeremias, Franziska R. Traube, Thomas Carell

**Affiliations:** ^1^ Institute for Chemical Epigenetics and Center for Nucleic Acid Therapies Department of Chemistry LMU Munich Munich Germany; ^2^ Research Unit Apoptosis in Hematopoietic Stem Cells Helmholtz Munich German Research Center for Environmental Health (HMGU) Munich Germany; ^3^ German Cancer Consortium (DKTK), partner site Munich a Partnership Between DKFZ and University Hospital LMU Munich Munich Germany; ^4^ Department of Genetics/Epigenetics Universität Des Saarlandes Saarbrücken Germany; ^5^ Medizinische Klinik und Poliklinik III Campus Großhadern LMU University Hospital LMU Munich Munich Germany; ^6^ Cluster for Nucleic Acid Therapeutics Munich (CNATM) Munich Germany; ^7^ Institute of Biochemistry University of Stuttgart Stuttgart Germany

**Keywords:** acute myeloid leukemia, Decitabine, DNA hydroxymethylation, DNA methylation, DNMT inhibition, epigenetics

## Abstract

Ten‐eleven translocation (TET) enzymes are critical epigenetic regulators, which oxidize the methylated cytosine nucleobase 5‐methyl‐dC (mdC) in the genome to 5‐hydroxymethyl‐dC (hmdC) in an α‐ketoglutarate‐dependent manner. Because the presence of mdC in the promoter region of a given gene silences its expression, this oxidation goes in hand with the reactivation of such silenced genes. In different highly aggressive cancers such as acute myeloid leukemia (AML) and glioblastoma, loss of TET enzyme function, and therefore reduced hmdC levels pave the way for tumor development. Impairment of TET activity can occur through metabolic inhibition, through loss‐of‐function mutations in TET genes themselves, and finally through suppression of TET‐expression via epigenetic silencing. Reactivation of TET enzyme expression represents a major aim of epigenetic cancer therapy. Here we show that the carbocyclic antimetabolite 5‐aza‐2′deoxycytidine (cAzadC), which is supposed to suppress the methylation of DNA during replication, leads to a substantial increase of TET2 expression and strongly increasing hmdC levels. We show that the treatment with cAzadC goes in hand with the broad reactivation of the cellular antitumor responses. With patient‐derived xenograft AML‐mouse models, we show that this translates into a strongly improved anticancer effect in vivo.

## Graphical abstract

The carbocyclic Decitabine analog (cAzadC) incorporates as an antimetabolite weakly into the genome of growing tumor cells, where it triggers a genome‐wide demethylation and a surprisingly strong hydroxymethylation of cytosine, which translates into an efficient in vivo antitumor (AML) effect.

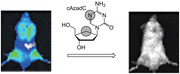

Epigenetic control of transcription is a complex process that involves the reversible modification of histone proteins, for example, via acetylation and methylation chemistry. In addition, it involves the chemical modification of genomic cytosine (dC) nucleosides, particularly in promoter regions [1, 2, 3]. On this genomic level, cytosines are methylated to 5‐methyl‐dC (mdC) by DNA‐methyltransferases (DNMTs) (Figure 1A), leading to reduced transcription of the corresponding genes [4]. Gene reactivation requires the oxidation of the methyl group in mdC to 5‐hydroxymethyl‐dC (hmdC) [5, 6]. This oxidation is achieved by α‐ketoglutarate (αKG)‐dependent TET dioxygenases [7, 8]. In cancer, where tumor suppressor genes are often found silenced, reduced TET activity and consequently reduced hmdC level are a hallmark [9, 10, 11, 12].

**FIGURE 1:**
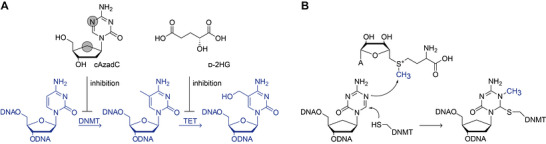
DNA methylation dynamics. (A) Depiction of the inhibitory cAzadC antimetabolite and the D‐2HG onco‐metabolite and methylation of dC to mdC by DNMT enzymes, followed by the TET‐induced oxidation of mdC to (hmdC). (B) Mechanism of DNMT inhibition by cAzadC.

In some tumors, such as many acute myeloid leukemia (AML) and glioma subtypes, neomorphic mutations of isocitrate dehydrogenases 1 and 2 (IDHs), which typically biosynthesize αKG from isocitrate, lead to the production of the onco‐metabolite d‐2‐hydroxyglutarate (d‐2HG), which can accumulate to millimolar concentrations in the tumor cells [13]. At these concentrations, d‐2HG inhibits the TET enzymes, which prevents the reactivation of genes that would otherwise stop uncontrolled cell growth [14, 15, 16]. Because efficient IDH inhibitors are now available, tumors that feature IDH mutations have a better prognosis [17, 18, 19, 20, 21, 22].

In many tumors, however, TET enzymes are epigenetically silenced [23, 24] and in these cases, IDH‐inhibitors are not expected to improve therapy. A rather unexplored approach to tackle this problem is to increase the expression of TET enzymes [25]. Here we show that this is indeed possible with the carbocyclic 5‐aza‐2′deoxy‐cytidine antimetabolite (cAzadC) compound shown in Figure 1 [26, 27].

We began the investigation by analyzing how well cAzadC would integrate as an antimetabolite into the genome of treated cells upon cell division. Once integrated, we knew that the compound would covalently react with the DNMT enzymes via the mechanism shown in Figure 1B. The resulting inhibition of the DNMT enzymes leads to a global reduction of the genomic methylation level, as previously shown by us for MOLM‐13 cells [27]. We investigated the ability of cAzadC to reduce mdC levels in different AML cell lines, treating Kasumi‐1, HL‐60 and for direct comparison again MOLM‐13, as well as patient‐derived xenograft (PDX) AML cells (AML‐491 [28]) for 72 h with cAzadC at concentrations of 0.5 and 1.0 µM. We used our stable isotope standard‐based UHPLC‐QQQ‐MS quantification method [29] in order to measure the levels of cAzadC in the soluble metabolite pool and in the genome. In addition, we quantified the levels of the epigenetic bases mdC and hmdC. For the experiment, we first isolated the nucleosides from the soluble cell fraction and from total DNA that was subsequently digested to nucleosides with a nuclease enzyme mixture. We then prepared a mix of synthetic, stable isotope labelled compounds cAzadC*, mdC* and hmdC* for co‐injection into the UHPLC‐QQQ‐MS system. For quantification, we determined calibration curves. This procedure allowed us to extract quantitative information from the MS‐signal intensities. The obtained data are depicted in Figure 2.

**FIGURE 2:**
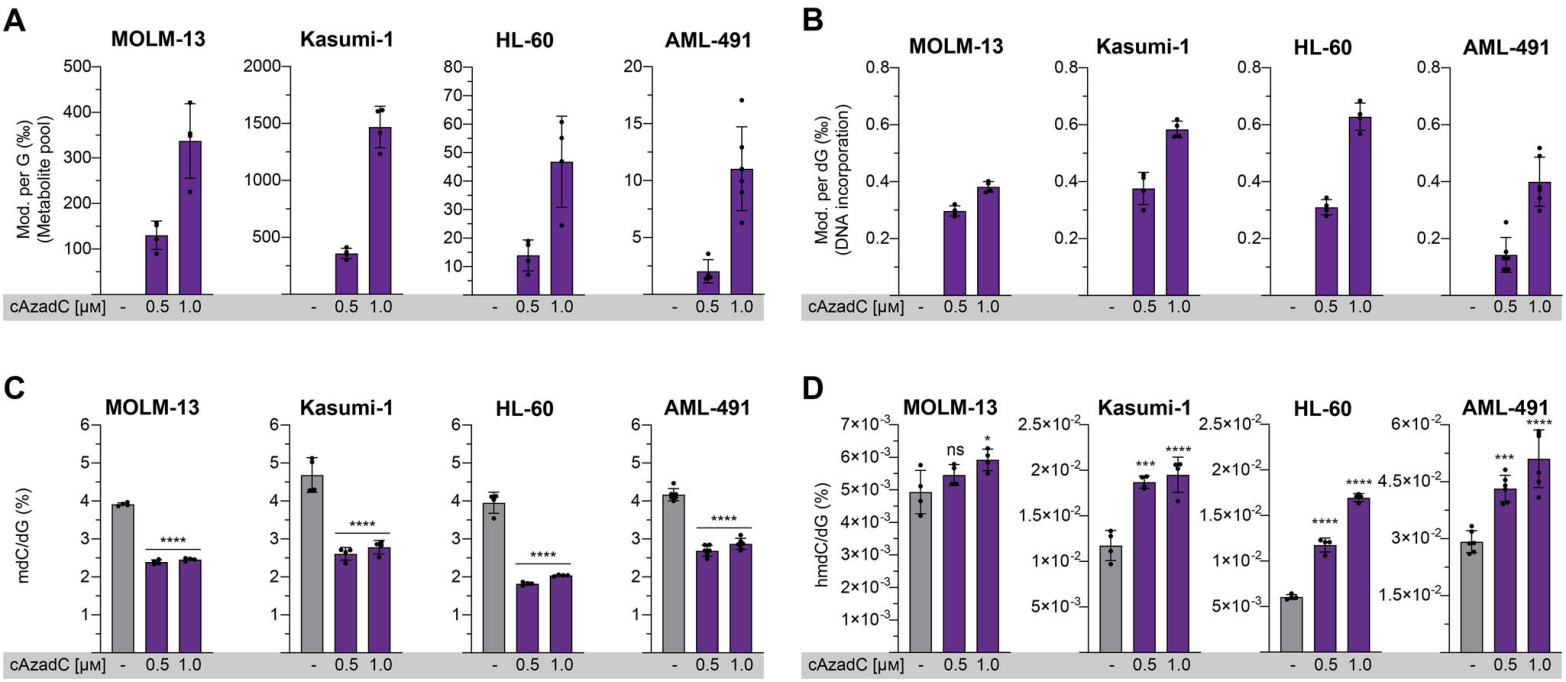
Metabolism and epigenetic effect of cAzadC. (A–D) Quantitative data about the amount of cAzadC in the soluble cell fraction (A), after genomic incorporation (B), and how treatment with cAzadC affects the levels of mdC (C) and hmdC (D). All data above are from cells harvested after 72 h of incubation with DMSO control, 0.5 or 1 µM cAzadC. The samples were proceeded as described in the methods and analyzed by UHPLC‐QQQ‐MS. The quantitative values were determined using heavy labelled ISTD and then normalized per hundred or thousand dG. Each dot represents the result from one biologically independent replicate. Statistical analysis was performed using ordinary one‐way ANOVA combined with Dunnett's multiple comparisons test (Ctrl. vs. treated samples); **** *p*_adj_ < 0.0001, *** *p*_adj_ < 0.001, ** *p*_adj_ < 0.01, * *p*_adj_ < 0.05, and ns = not significant.

Based on literature data, we expected that replacement of the ribose by a cyclopentane unit would negatively affect the ability of cAzadC to be integrated into the genome [30]. This is indeed observed. While the metabolite pool accumulated significant amounts of cAzadC even after 72 h (Figure 2A), the amount of genome‐integrated material remained very low, but clearly detectable (Figure 2B). In the AML cells lines and in the PDX AML‐491 cells, we observed an incorporation level of about 0.5 ‰ per dG at 1 µM dosing, which corresponds to the presence of about one million cAzadC molecules in the genome. This is very low compared to other antimetabolites, but enough to see an improved cell death after a cellular division (72 h), as represented by flow cytometry‐based apoptosis data in MOLM‐13 cells (Figure S1A). As previously shown, the integration is sufficient to deplete the entire DNMT1 pool [31, 32].

Despite low integration levels, we saw in all cell lines, and particularly in the PDX AML‐491 cells, a significant reduction of the mdC levels from about 35% (AML‐491) to 55% (HL‐60) (Figure 2C), showing that the low level of integration does not impair the epigenetic effect.

In contrast, we detected substantially increased levels of hmdC, ranging between 15% (MOLM‐13) and 300% (HL‐60) increase (Figure 2D). Importantly, also in the PDX AML‐491 cells, the hmdC levels increased by 66% from 0.03% to 0.05% relative to dG. This surprising observation might be explained by profound changes in gene expression, potentially caused by global genomic demethylation of the genome and thereby increased *TET* expression levels, but clearly requires further investigation.

To this end, we checked for global transcriptome changes analyzed by RNA‐seq in MOLM‐13 cells and observed a small, but significant increase in *TET2* gene expression levels (Figure 3A). In addition, and in line with downregulation of the αKG‐depleting transaminases BCAT1 and BCAT2 [33] (Figure 3A), also intracellular αKG‐levels were increased to a small, but significant extent (Figure 3B). The combination of increased *TET2* expression and αKG‐levels could therefore explain the moderately higher TET activity as indicated by the small increase in hmdC in MOLM‐13 cells after cAzadC‐treatment (Figure 2D). Among the other genes monitored, it is noteworthy that growth‐inhibiting p53 signaling was highly upregulated, while cancer‐promoting MYC signaling was highly downregulated (Figures 3C and S1B) which is in line with the idea that the reduction of methylation levels creates a strong antiproliferative response (Figure S1C). Interesting and potentially clinically relevant is the observation that the antiapoptotic protein BCL‐2, often found overexpressed in leukemic cells, and therefore in the clinic inhibited by treatment with Venetoclax [34], was significantly downregulated after cAzadC treatment alone (Figure 3A). In line with these observed transcriptomic changes, cAzadC‐treated MOLM‐13 cells underwent apoptosis (Figure S1A).

**FIGURE 3:**
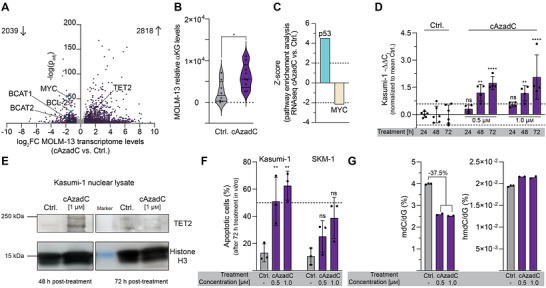
Addressing the hallmarks of cancer in MOLM‐13 cells with cAzadC treatment and effect of cAzadC on TET2 function in different AML cell lines. (A–C) MOLM‐13 cells were treated as indicated once with 0.5 µM of cAzadC for 72 h and the effects were compared to DMSO‐treated Ctrl. (A) Volcano plot of transcriptomic changes. RNAseq analysis with *n* = 3 biologically independent replicates per condition. The significance threshold for differential expression was set to −log(*p*_adj_) > 1.3 and |log_2_FC| ≥ 0.5849 (|fold change| ≥ 1.5) treatments. (B) Relative αKG levels determined by a fluorescence assay after treatment displayed as violin plot (including 25th percentile (lower border), median (middle border), and 75th percentile (upper border)). Statistical analysis was performed using two‐sided *t*‐test. * *p*‐value_j_ < 0.05 and ns = not significant. (C) Results of the pathway enrichment analysis using ingenuity pathway analysis [37]. (D) *TET2* gene expression levels in Kasumi‐1 cells following treatment with 0.5 µM or 1.0 µM cAzadC, as determined by RT‐qPCR. *TET2* mRNA expression was normalized to *GAPDH* as the housekeeping gene for Δ*C*_t_ calculation. For ΔΔ*C*_t_ analysis, *TET2* expression levels were normalized to the mean control level at the respective time point. Statistical analysis was performed using two‐way ANOVA combined with Tukey's multiple comparisons test and results of treated samples compared to DMSO‐treated control for each timepoint are displayed. **** *p*_adj_ < 0.0001, *** *p*_adj_ < 0.001, ** *p*_adj_ < 0.01, * *p*_adj_ < 0.05, and ns = not significant. (E) Immunoblot results for TET2 levels in Kasumi‐1 cells after treatment with 1 µM cAzadC. Histone H3 was used as a loading control for the amounts of nuclear lysate. (F) Results of the flow cytometry‐based apoptosis assay as previously described [35]. Kasumi‐1 and SKM‐1 cells were treated with 0.5 or 1.0 µM of cAzadC for 72 h. Statistical analysis was performed using one‐way ANOVA combined with Šídák's multiple comparisons test to compare within each AML cell line DMSO‐treated control to cAzadC‐treated samples. **** *p*_adj_ < 0.0001, *** *p*_adj_ < 0.001, ** *p*_adj_ < 0.01, * *p*_adj_ < 0.05, and ns = not significant. (G) UHPLC‐QQQ‐MS results for mdC and hmdC in SKM‐1 cells after 72 h cAzadC‐treatment. (B,D,F,G) Dots represent biologically independent replicates and for (B,D,F,G) bars represent mean; error bars S.D.

Since MOLM‐13 cells only showed a small increase in hmdC compared to other AML cell lines, we subsequently analyzed how TET expression levels were affected in Kasumi‐1, which showed a substantially higher upregulation of hmdC after cAzadC treatment (Figure 2D). Here, we observed a time‐dependent and significant increase in *TET2* gene expression (Figure 3D), while the expression levels of the other TET enzymes (TET1 and TET3) were not significantly affected (Figure S2A), which is consistent with the particularly prominent role of TET2 among the TET enzymes in the hematopoietic context. Next, we checked whether the increased *TET2* gene expression levels in Kasumi‐1 cells after cAzadC treatment also translate into higher protein expression levels. Following 48 h of 1 µM cAzadC treatment, Kasumi‐1 cells exhibited a pronounced increase in TET2 protein levels relative to DMSO‐treated control (Figures 3E and S2B). Interestingly, despite persistently elevated *TET2* mRNA expression (Figure 3D) and sustained high genomic hmdC levels at 72 h post‐treatment (Figure 2D), TET2 protein abundance was again diminished at this later time point. Since more than 50% of Kasumi‐1 cells underwent apoptosis after 72 h of cAzadC exposure (Figure 3F), we assume that the decline in TET2 protein levels is not due to transcriptional downregulation but rather reflects apoptosis‐associated impairment of protein biosynthesis and enhanced protein degradation. In contrast, the elevated hmdC levels likely persist because of the relative stability of DNA modifications within the investigated time window.

Since enhanced TET2 activity has previously been linked to favorable responses to DNA hypomethylating therapies [35, 36], we asked whether loss‐of‐function *TET2* mutations impair AML cell sensitivity toward cAzadC. Consistent with this hypothesis, the TET2‐mutant AML cell line SKM‐1 exhibited a markedly weaker apoptotic response following cAzadC treatment (Figure 3F). Nevertheless, UHPLC‐QQQ‐MS analysis confirmed genomic incorporation of cAzadC (Figure S2C) also in SKM‐1 cells, and mdC levels were reduced comparable to other AML cell lines tested (Figure 3G), excluding resistance mechanisms upstream of DNMT inhibition. Of note and in line with the TET2‐deficient background of SKM‐1 cells, hmdC levels remained largely unchanged upon treatment (Figure 3G). While these observations must be interpreted cautiously regarding the pro‐apoptotic role of reinforced TET2 expression due to the distinct genetic backgrounds of AML cell lines, our findings support a model in which increased TET2 expression and catalytic activity enhance the therapeutic response to cAzadC.

To investigate how patient‐derived AML‐491 cells would react, we first quantified the levels of *TET2* mRNA after treatment. In these studies, we included the clinically established AzadC compound (Decitabine) [38, 39] as a reference. While the Decitabine treatment yielded highly inconsistent *TET2* expression changes, the cAzadC compound increased the *TET2* expression robustly in a dose‐dependent fashion more than 20‐fold at the highest concentration of 3 µM (Figure 4A). This is a dramatic effect and can only be explained by assuming that the expression of the epigenetically acting enzyme TET2 is indeed tightly repressed by DNA methylation and that the global demethylation, caused by cAzadC, leads to an unleashing of its expression. Whatever the exact reason may be, the data show that the complex genetic rewiring processes that are associated with global epigenetic demethylation caused by cAzadC leads to a substantial increase of the amount of TET2. This, in combination with higher αKG levels is suggested to be responsible for the increasing hmdC levels.

**FIGURE 4:**
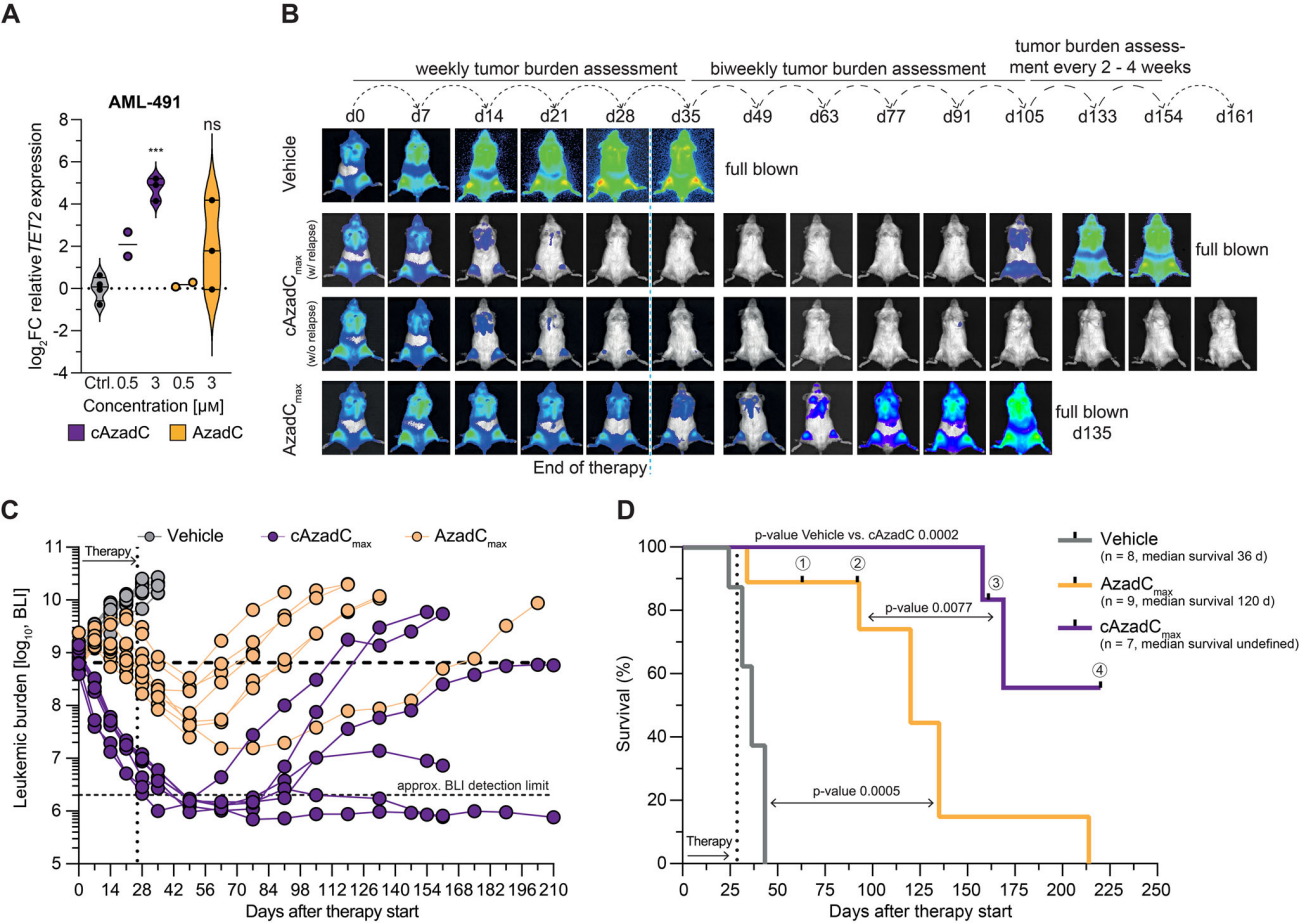
Effects of cAzadC treatment compared to AzadC (Decitabine) treatment and vehicle in vivo. (A) log_2_ fold‐change of TET2 expression levels in AML‐491 PDX cells after ex vivo treatment with cAzadC compared to AzadC. (B–D) NSG mice were transplanted i.v. with luc‐positive AML‐491 PDX cells. Leukemic burden was monitored by repeated bioluminescence in vivo imaging (BLI). At reaching a total flux of 7 × 10^8^ Photons/second, mice were treated with 40 mg/kg cAzadC (cAzadC_max_) or 0.25 mg/kg AzadC (AzadC_max_) or vehicle as control i.p. for 5 days a week for 4 weeks. Representative BLI images (B) and quantification (C), where each curve represents one mouse. (D) Kaplan–Meier curves of mice treated with vehicle (*n* = 8 of 3 independent experiments), AzadC_max_ (*n* = 9 of 3 independent experiments) and cAzadCmax‐treated (*n* = 7 of 2 independent experiments). Median survival after therapy start: vehicle (grey): 36 d, AzadC_max_ (orange): 120 d, and cAzadC_max_ (violet): undefined. Log‐rank (Mantel‐Cox) test.

Because increasing hmdC levels and higher TET2 activities were in the past firmly associated with reduced tumor growth [36, 40] and this finding was strongly supported by the results obtained in this study, we reasoned that in comparison to Decitabine, which despite substantially higher integration into the genome showed a substantial weaker impact on TET2 expression and activity in all AML cell lines tested (Figures S2 and S3), cAzadC would show improved antitumor activities also in an in vivo setting.

In order to investigate this, we performed an in vivo treatment experiment in a mouse xenograft model. We injected luc‐positive AML‐491 PDX cells into NSG recipient mice, which are immunodeficient to accept engraftment of primary human cells, and measured tumor outgrowth by following their bioluminescence (BLI) in vivo. Twentyone days after injection (referred to as d0), mice were treated for 4 weeks with either cAzadC, Decitabine or vehicle. Knowing about the different genomic integration kinetics of cAzadC and Decitabine, we decided to perform the comparison at the respective maximum tolerated doses determined previously in a toxicologic study [27]. There, an over 100‐fold better tolerance was observed for cAzadC compared to Decitabine. While this limited us to 0.25 mg/kg per day over 4 weeks for Decitabine, we were able to increase the dose to 40 mg/kg per day for cAzadC. The comparison at these maximum tolerated doses showed that, consistent with our speculation, a more rapid decrease in leukemic burden is observed with cAzadC (Figure 4B,C). The tumor reduction also lasted longer, for even up to 3 weeks after the end of treatment (Figure 4B,C). Interestingly, the re‐growth of tumor cells at the end of therapy was greatly reduced in both Decitabine and cAzadC‐treated mice compared to AML‐491 cells of the control that have not undergone treatment, but more pronounced for cAzadC (Figures 4C and S4). In two mice, leukemic stem cells (LSCs), expected to be the source of relapse, remained functional as indicated by the resumption of leukemic cell proliferation. In one mouse, leukemic cell proliferation resumed, but stopped before reaching again the BLI values at the beginning of the therapy. In another mouse, proliferation initially resumed, but then stopped and dropped without further treatment below the BLI detection limit, suggesting exhaustion of leukemic cells. Remarkably, in two mice, the leukemic burden remained below the detection limit until they had to be sacrificed due to leukemia‐unrelated end‐points, indicating that LSCs were effectively eradicated (Figure 4C). In contrast, Decitabine treatment at the maximum tolerated dose failed to eradicate LSCs, leading in all mice to resumption of proliferation. Despite a significantly increased survival time, all Decitabine‐treated mice had to be sacrificed prematurely due to high leukemic burden. Overall, we observed a significant increase in survival of cAzadC treated mice, not only in comparison to control‐treated mice, but also compared to mice treated with Decitabine at the maximum tolerated dose (Figure 4D).

In summary, our findings indicate that reactivation of TET2 activity represents a major contributor to the antileukemic efficacy of cAzadC and may explain its superior therapeutic effects compared to Decitabine. Although both compounds induce DNA‐DNMT crosslinks, accompanied by DNA hypomethylation and DNA damage, Decitabine showed substantially higher genomic incorporation (Figure S3) and is therefore expected to generate at least similar, if not greater, levels of DNMT trapping. Nevertheless, Decitabine induced considerably weaker TET2 reactivation and antileukemic activity compared to cAzadC. These results strongly suggest that the enhanced therapeutic activity of cAzadC is not merely a consequence of DNA‐DNMT crosslink formation, but is importantly linked to its capacity to restore TET2 function.

## Author Contributions

**Maike Däther**: investigation, validation, visualization, formal analysis, methodology. **Elsa Peev**: investigation, methodology, validation, visualization, formal analysis. **Annika Fröhlich**: investigation, methodology, validation, visualization, formal analysis. **Binje Vick**: investigation, methodology, validation, visualization, conceptualization, formal analysis. **Sogol Fatourechi**: investigation, methodology, validation, visualization, formal analysis. **Gilles Gasparoni**: investigation, methodology, validation, visualization, formal analysis. **Matthias Heiss**: investigation, methodology, validation, conceptualization, formal analysis, project administration. **Corinna C. Pleintinger**: investigation, methodology, validation, formal analysis. **Emmanuel Asu Bisong**: investigation, methodology, validation, formal analysis, resources. **Hans Hurmiz**: investigation, methodology, validation, formal analysis, resources. **Davide Guglielminotti**: investigation, methodology, validation, formal analysis, resources. **Yasmin V. Gärtner**: investigation, methodology, validation, visualization, formal analysis. **Tina Aumer**: investigation, methodology, validation, visualization, formal analysis. **Karsten Spiekermann**: funding acquisition, project administration, supervision. **Jörn Walter**: funding acquisition, project administration, supervision, conceptualization. **Irmela Jeremias**: conceptualization, funding acquisition, project administration, supervision. **Franziska R. Traube**: conceptualization, investigation, funding acquisition, writing – review and editing, methodology, validation, visualization, formal analysis, project administration, supervision, data curation. **Thomas Carell**: conceptualization, funding acquisition, writing – original draft, project administration, supervision.

## Conflicts of Interest

The authors declare no conflicts of interest.

## Supporting information



The authors have cited additional references within the Supporting Information [29, 40, 43, 44, 45, 46, 47, 48, 49].
**Supporting File**: anie73204‐sup‐0001‐SuppMat.pdf.

## Data Availability

The RNAseq data of 0.5 µM cAzadC or AzadC treated MOLM‐13 cells were deposited at the gene expression omnibus (GEO) repository [41, 42] with the dataset identifier GSE225154. All other data is available in the Supporting Information.
